# Urinary Deposits; Their Diagnosis, Pathology, and Therapeutical Indications

**Published:** 1858-09

**Authors:** 


### NORTH AMERICAN

### MEDICO-CHIRURGICAL REVIEW.

SEPTEMBER, 1858.

### Analytical and Critical Reviews.

Art. I.—*Urinary Deposits; their Diagnosis, Pathology, and Therapeutical Indications.* By Golding Bird, M.D., F.R.S. Fifth Edition. Edited by Edmund Lloyd Birkett, M.D., Caius Coll. Canterb., Fellow of the Royal College of Physicians, etc. etc. etc. 8vo. pp. 494. London: John Churchill, 1857.

The name of Golding Bird is to be added to the list of physicians who have died of diseases which have been the subjects of their own peculiar study. Of this kind of coincidence, Corvisart and Laennec are better known examples, and the list might be further extended. It is scarcely possible that the habit of fixing the attention upon a given organ, as the lungs or kidney, should predispose that organ to disease; or yet further, should actually create a pathological condition. It is more possible that the consciousness of the weakness of an organ, or of hereditary tendencies to a particular malady, should give specific direction to the studies of a physician. It is most probable, after all, that the coincidence alluded to is entirely accidental.

Apart from the fact which we have just mentioned, the early death of the accomplished author of the volume before us was calculated to excite the sympathy of all men who respect great talent, and esteem that lofty industry which is fruitful of good results, and never weary of well-doing. In the words of his editor—"His time and strength, both of body and mind, were given to the profession, and he reaped, as a natural consequence, its highest rewards; but those who knew him best, felt that he had brighter hopes beyond, which supported him through a life of sickness, and comforted him at its close."

The volume under review is the principal literary result of his labors in a branch of medicine which he undoubtedly has done more to make familiar to the profession generally, than even some of those who, like Prout and Becquerel, have been more productive of highly original results. The first edition of this work was published in 1843, and was chiefly founded upon papers and lectures, which originally appeared in the *Medical Gazette* and in *Guy's Hospital Reports.* This edition was reprinted in this city, in 1844, by Lea & Blanchard, and has passed through several later editions, both here and in London. Of the copy before us, the fifth English edition, we have as yet had no reprint here. When such a one is published, we hope to witness some effort towards imitating the luxury of type and illustration which distinguish the medical manuals of the English house of Churchill from the Cisatlantic reprints.

Let us pause here to state that we are no advocates of the plan of republishing foreign books without the sanction of the author, and without compensation for weary hours, stolen from the turmoil of professional life, to devote to the tasks of authorship. This is bad enough; but we might, at least, afford to gild the bitter pill. It is hard, indeed, to steal a man's plate, but to melt it into ugliness and past recognition, is rather worse. It would, indeed, be a wise English author who would know his own book in its Yankee costume. As to American editions of those aristocratic Sydenham and Cavendish Society books—they are enough to set one's paper-cutter on edge. When shall we learn that men's brains are not umbrellas—are not everybody's property?

Happily for us, we propose to review the last, the fifth English edition, with the editorial notices of Edmund Lloyd Birkett. This stout and handsome octavo manual is the latest summary of our knowledge in regard to the Urine. It now lies upon our table, side by side with a dozen volumes which record the progress of this branch of medicine since it was derived from the Arabs, and assumed such undue importance in the hands of the Urine Casters in the 15th and 16th centuries.* These gentry seem to have been the homœopaths of their day—nay, they were, to a minimum extent, better than these; for, in place of giving little doses, they gave none at all—beyond which degree of perfection it does not seem to us that the globulist can go, even with the aid of a world grown grayer by three centuries. In despite of the war to the scalpel which the regular profes-

* "If thou couldst, Doctor, cast
The water of my land, find her disease,
And purge it to a sound and pristine health."
MACBETH.

sion waged against these quacks, the practice of Uroscopy not only continued to be popular, but so fashionable, also, that at some of the German Courts an official examiner daily inspected the water of the reigning sovereign. Some of the books of these personages are amply amusing, and it is interesting to notice the succession of them, under divers names, until they appear in yellow-covered descendants of to-day, as books of advice to the young, etc. etc. The Urine Casters no longer write books, and, in fact, are with us rather rare curiosities. Uroscopy is no longer profitable, and we have, in its place, the advertising Doctor, who, however, in despite of his yellow tracts, is deserted by the muses, who once aided the Urinary Doctor. Indeed, many old books on Urine Casting contain verses complimentary and flattering, from the patients of the lucky Doctor. M. Davach de la Riviere appears to have been highly favored in this way. One of his clients invites us to

"Courez donc chez Davach si vous voulez guerir,

Il sçait, voyant l'urine, empecher de mourir."

Certainly a desirable consummation, but, unluckily, no longer attainable, since, as another of his poetical patients informs us,

"Henreux sont les mortels dans le siécle ou nous sommes,

Davach par ses écrits soulage tous les hommes."

Notwithstanding the volume before us, and many others, more or less valuable, we fancy that the profession, in general, still regards this study as unnecessary to complete a medical education. Indeed, we have little hesitation in asserting, that a large part of the mass of medical men are incapable of distinguishing the various urinary sediments, by the eye or the microscope. Yet, if any branch of medicine is, of all branches, the least fitted to become the prey of mere specialists, it is this one—since a good knowledge of the urinary fluids, and of the means for their study, is, we fear not to add, as much an essential to the real physician, as a knowledge of pulmonary complaints and of the uses of the stethescope. When this is to become a recognized part of every complete system of medical training, we cannot say. Already, in some examinations abroad, the candidate is required to use the microscope, and to ascertain with it the character of urinary deposits, of tissues, etc. We are a little fearful, that now and then, among the graduates of this country, there would be found young men of excellent theoretical attainments, but quite unable to tell an écraseur from a microscope.

As an example of this sort of ignorance, or of far worse, we recall an incident which chanced to us a few years ago, and which very well shows the utility of the study we have been recommending, as well as the danger of neglecting one of the plainest and most valuable precepts with which it has enriched the science of surgery.

Some years ago, a patient applied to us for relief from certain vague sensations of uneasiness, and on account of an inexplicable feeling of fatigue which every exertion brought upon him. We discovered that he was suffering from sacchuria. During two years his case was more or less before us, and remained very much the same, neither better nor worse. For reasons best known to himself, he chose to conceal its existence from his family. A year or two afterwards this gentleman died very suddenly, after an operation of no very formidable nature, but which no sane surgeon would have performed had he supposed his patient to be suffering from diabetes. No examination of the urine was even thought of. The symptoms mentioned, were ascribed to the malady which the operation was to relieve, and the patient himself, having adopted a similar view, had failed to mention the existence of a constitutional disease, which he had ceased to regard as of moment. The neglect of a precaution which should always precede an operation of any importance, cost the patient his life, and no doubt greatly puzzled the unlucky operator.*

The excellent little text-book of our author has been so long before the medical public that it would be time lost to analyze its contents very fully; but there are some portions of it which are sufficiently novel, owing to recent additions, to exact this at our hands, and there are others which are invitingly open to comment and discussion.

The first chapter of the volume is intended to assist the student, by giving him a general view of the composition of normal urine, and by teaching him the readiest methods of analyzing this complex fluid, and acquiring a knowledge of its various physical characters. The main object is, of course, to make these examinations possible for men who are actually engaged in business. Something has been done toward attaining this desirable result by the application of volumetric analysis to the determination of the quantities of the urinary compounds. It is neither possible here, nor requisite, to state the details of these operations; but how successfully they have been used may be seen by a reference to the recent prize essay of Dr. Hammond. The best statement of these methods is to be found in the first two numbers of Dr. Beales' new journal, *The Archives of Medicine*, where they are so minutely detailed as to make it possible to conduct them with success. We scarcely can say as much of the quoted description of them in the work before us.

Besides this method, which may be made a very accurate one, Heller

* The remarks of Bence Jones upon this subject refer chiefly to the existence of Bright's Disease. They admit of a more general application.

has proposed another which answers well enough for most clinical purposes, although it can never be applied to the collection of physiological facts. As every chemist knows, when urine is partially evaporated, nitric acid will throw down its urea. Without evaporation a strong acid will precipitate the uric acid, and when the urine is warmed will act upon its pigments. Again, nitrate of silver precipitates the chlorine of the common salt in urine, and baryta salts produce insoluble sediments with the sulphuric acid of this fluid. Now, as we have hinted, these reactions are made accurate use of in volumetric analysis. If, however, we do not require perfect results, we may use these reactions to good purpose by always working with known, and the same quantities of the urine, and with test solutions of constant strength. By this means, and by often testing normal urine, the eye at length becomes so accustomed to estimate quantities from the bulk and appearance of precipitates, that it is usually easy to detect any important deviations from the healthy standard. Having no intention of ignoring the more accurate means, we still think that, by a little practice, the rough analysis just sketched may be made of much clinical use. Any one who has observed the accuracy with which a skilled hand at blow-pipe analysis will guess at the per centage result from the size, etc., of the bead or globule left upon the charcoal, will scarcely be surprised that we should venture to advocate the limited use of processes to all seeming so inaccurate.

At the close of the first and second chapter the editor refers to the value of the microscope in the study of the urine, in health and in disease. We may as well state here our conviction, that no man can be a completely accomplished physician who neglects its aid, and that without it some diseased conditions of the urine are utterly out of his medical view. It is quite unnecessary to possess an expensive instrument for these purposes. Indeed, a microscope of sufficient power can easily be had for from twenty-five to forty dollars.* The student will find the general directions at the close of Chapter II. of great utility. Our author's remarks upon the general worthlessness of the urinometers sold in the shops we heartily endorse.

The third chapter treats of the chemical physiology of the urine, and, after discussing its acidity and classifying its ingredients, passes on to the account of each one of them, commencing with urea, the most important of all. One point arrests us in regard to the acidity of the urine. The editor believes, with Bence Jones, that the acidity of the urine holds a direct relation to that of the stomach. Thus, the urine is stated to be most acid when the contents of the stomach possess the greatest acidity, and

---

* Such glasses are to be had of Mr. Queen and of Mr. McAllister, of this city.

*vice versa. Immediately before each meal* the urine always showed the greatest acidity. Now we have long labored under the idea that, just before most of our meals,—when these were separated by some hours,—the stomach either had no contents, save mucus, or had contents which were quite neutral until the irritation of new food called forth a fresh supply of gastric acid from the secreting glands. Again,—and here we reduplicate the puzzle,—if the amount of acid in the stomach regulates that in the urine, are we to regard the two acids as identical? and if not, what part does the intervening alkaline blood play? and if the acids are not the same, must we borrow a therapeutist's term, and state that the presence of acid in the stomach acts as an alterative upon the quantity of another acid present in the urine? Our author and his editor do not mention the experiments of Claude Bernard upon this subject. He has shown definitively, as it appears to us, that a nitrogenous diet, persisted in for a time, invariably yields an acid urine, as does the mere metamorphosis of tissues in starving animals; while, on the other hand, a non-nitrogenous diet gives rise to an alkaline state of the secretion in question. (See Bernard, Leçons de Physiologie; Semestre d' été, 1855.)

A considerable share of this chapter is taken up with the physiological relations of urea and uric acid. The editor has done very little for this portion of the book. Indeed, neither he nor the author have treated it so fully as its great importance demands, and both of them have entirely ignored the papers which have been written in this country, and which, although not numerous, are otherwise well entitled to notice. Irrespective of this culpable omission, the arguments against the views of Prof. Liebig are excellently stated, although in some points defect of research has committed the author or editor to certain rather doubtful statements. It is easy to indicate the main points in this question without too tediously elaborating our views.

Since urea and uric acid are both found in the urine of man, and since the one or the other is always to be detected in the urine of animals, it was supposed by Liebig that these two important excretives held to one another a remarkable relation. "He believes that when, in the exhausted tissues containing protein, (*i.e.*, albuminous structures,) the vital force is no longer able to resist the chemical action of the oxygen which is conveyed to them in arterial blood, it combines with their elements, and forms products, among which uric acid is the most important. Thus the elements of one atom of the essential ingredients of all muscular and fibrous tissues, (protein,) with ninety-one atoms of oxygen, are equal to the elements of uric acid, carbonic acid, and water. If, then, sufficient oxygen and water be conveyed in the arterial blood, the greatest part of the uric acid may be converted into carbonic acid and urea, so that the effete nitrogenized elements of the tissue reach the emunctories in a soluble form, a condition necessary for their ready excretion." Now, as both urea and uric acid exist in the blood, it becomes plain that, on the above-stated hypothesis, the larger the amount of oxygen circulating in the blood, the more complete will be the conversion of uric acid into urea. Thus, if the supply of oxygen is abundant, and the circulation active, the urea will be increased, and the acid lessened. Accordingly, the urine of serpents contains no urea, and a large amount of uric acid. Again, the urine of lions, tigers, dogs, etc., all active carnivoræ, does not contain any appreciable quantity of uric acid, and here the oxidation of tissues is supposed to be so complete as to transform all of the uric acid into this more soluble substance. If, then, the relative activities of various animals, as enabling them to take in more oxygen, be of moment in this connection, it follows that in man, whose urine contains both urea and uric acid, exercise should increase the oxidation of tissues, and thereby augment the urea, while inactivity should produce a directly opposite result. Accordingly, many researches have been instituted to solve the problem from this point of view. The results of Dr. Hammond's researches certainly seem to indicate the correctness of Liebig's ideas. Dr. H. found that exercise invariably increased the urea, and lessened the amount of uric acid. Unfortunately, other observers, especially Draper, are unable, after like researches, to support his results. Prof. Lehman has also shown that exercise increases the actual amount of urea in the urine, but he has not pointed out its varying relation, in this case, to the quantity of uric acid.

As an accessory to Liebig's theory, it has been stated that all highly carboniferous food should diminish the urea, and increase respectively the uric acid; because, when the body is loaded with materials easily oxidized, like the kind of diet alluded to, too little oxygen remains unemployed to effect the conversion of the uric acid into urea. This part of the question has also been answered in many ways. Thus Lehman has shown that a vegetable diet, and one quite free from nitrogen, decreases, and animal diet increases the amount of uric acid. Also, that the urea increases and decreases in a similar manner under these same circumstances. Dr. Frick, in stating the argument, in his book on *Renal Deposits*, thinks that these observations should be thrown out as evidence, because they stand alone,—a singular reason,—and because the whole amounts of the various excretives, as stated by Lehman, are so large as to excite distrust. He himself so entirely accedes to Liebig's views, that he thinks if man were in perfectly normal conditions, he would secrete nothing but urea, whence it would be clear enough that our first father, at least, did not suffer from the calculous diseases which, along with other plagues, his misdemeanors have entailed upon us. In confirmation of Liebig's views,

Dr. Frick also states that those convicts in the Baltimore penitentiary who led sedentary lives always excreted a great excess of uric acid, and that in proportion to the amount of exercise which they took was the decrease of this compound, and the increase of the urea. He also states that, when he gave cod liver oil to one of the active class, the urea fell off, and the uric acid increased. Dr. Frick is further of opinion that purgatives increase the urea excreted, by carrying off food and bile, which otherwise must have been oxidized in the body, and so have deprived the uric acid of the oxygen necessary to form from it urea.

Beyond these facts, if facts they all be, we have but little evidence of the presumed chemical relationship of urea and uric acid. We are not able, however, to go so far as our author, and state that *we have no proof of any kind* that uric acid is a necessary transition formation between protein compounds and urea, since Wohler and Frerichs have shown that the introduction of uric acid into the veins, or the primæ viæ, is followed by an increase of the urea and oxalate of lime in the urine—a decomposition which can be artificially imitated by acting upon uric acid with peroxide of lead.

Perhaps, however, there is nowhere else a piece of evidence for this theory so strong as one furnished by Dr. Hammond. (*Am. Journ. Med. Sci.*, Jan. 1855, p. 119, *et seq.*) This observer examined the urine of a black-snake, and satisfied himself that it contained no urea, and an abundance of uric acid. He then introduced into the jar which held the snake, a large amount of oxygen gas. This was repeated thrice a day for a week. The gas greatly excited the snake, but he became sluggish when the supply was withdrawn. The excrement passed during this time contained an abundance of urea as well as of urates.

The opponents of Prof. Liebig's theory have made out a very strong case against his views; and certainly we can in no way comprehend, from his point of sight, why the very active bird and insect, with fluids rich in oxygen, should excrete only uric acid like the inactive, slowly-breathing serpent. The researches of Magendie, Lehman, and Boussingault, cast further doubt upon the views of Liebig, and we are only able at last to say, with an approach to certainty, that both urea and uric acid are products of the disciplined decay of nitrogenous tissues—that one can replace the other completely in different animals, but that we are still in the dark as to the exact role which they play in relation to one another, and to the retrograde changes of tissues where both are found in the urine at once, as in man.

Upon the whole, after carefully reviewing the evidence, we incline to think that, while urea and uric acid are nearly connected links in the chain of regressive metamorphosis, and while uric acid is convertible into urea within and without the body, we are not as yet in possession of all the conditions which make this change easy or possible. Certainly the amount of oxygen present is not the sole governing agency.

The physiology of the urine is further dealt with by our author under the headings of the various remaining excretory compounds, as lactic acid, creatine and creatinine, hippuric acid, and butyric acid.

As regards the coloring matter of the urine, Bird makes out but little that is satisfactory. Simon thought it was hæmaphæin, the pigment of blood serum. Prout supposed that two urinary pigments existed. It is the halophyle of Berzelius, and the uroxanthin of Keller, who believes that, when oxidized, two other coloring matters arise from it—namely, uroglaucin, and uri-rhodin. These our author states to be identical with what he has called purpurine. If we remember aright, Keller also distinguishes a second normal pigment, which he terms urophœin, and to which he ascribes both the smell and reaction of the urine of health. It is easy to see, from this brief summary, how just is the statement of Lehman, that we know nothing at present of these singular compounds. We, ourselves, are inclined to think that every pigment in the body is due to changes in the blood corpuscles; and so far, indeed, as the urine goes, the evidence in favor of this view is very strong.

Ammonia and the fixed salts of the urine last occupy our author's attention. He next proceeds to classify the deposits which occur in urine; a portion of the volume demanding neither quotation nor criticism.

Chapter fourth is devoted to what our author terms lithic uria; and, although it has been objected that the deposit is more rarely a disease than a mere symptom, the terms lithic uria, oxaluria, etc., seem to be so convenient, and to have gained so firm a foothold, that it is useless to quarrel with them even if it were desirable. The chapter before us is a most admirable summary of our present knowledge upon the subject of lithic acid. If our author and his editor had taken the trouble to glance at the journal-literature of this country, they would have acquired some additional knowledge of some of the phenomena connected with the crystalline forms of uric acid; though, excepting this, we can only praise the clearness and completeness with which the subject matter is handled. As usual, the editor has failed to fulfill his task, and we find no allusion to the singular deposits of uric acid so often found in the kidneys of children at birth. Again, when examining the various means of rendering the urine alkaline or acid, we hear nothing of the valuable information upon these points to which we have before alluded, and which teaches us that a persistent vegetable diet will, in the end, cause the urine to exhibit an alkaline reaction, while animal food has a directly opposite effect. We are also struck with the fact that, after admitting that the exhibition of benzoic acid does not affect the quantity of uric acid present in the urine, our author proceeds to tell us, in the next page, that benzoic acid does limit the quantity of uric acid to be found in the fluid in question.*

The succeeding articles upon uric oxide and cystine, are of but little interest to the practical physician. We are struck, however, with the comparative rarity of cystine, as a urinary deposit, in this country; for, although we have repeatedly sought for it in the urine of chlorotic females, we have never met with it, either in these cases or in any of the numerous examinations of this fluid which we have made during the last five years. Dr. Frick, of Baltimore, has met with but one case, and we know of no record of any in our home journals.

The next subject of general interest which attracts our notice is the chapter upon the chemical pathology of oxalate and oxalurate (?) of lime. We confess that we are disposed to accept here the author's term oxaluria, and admit, at the same time, the existence of a particular pathological state, with features well enough marked to entitle it to this distinction. The editor of Dr. Bird has very well defended his author against those who do not believe in the existence of any disease entitled to be named oxaluria, from what they regard as but one symptom of several diseased conditions. We ourselves are willing to go beyond Dr. Bird and his editor also. In most of the cases where oxalic acid occurs largely, or even steadily, in the urine, there is a peculiar state of the general system, which we take to be a result of the existence of oxalic acid in the blood or tissues, or both. Whether it is generated in the blood itself or in the stomach is of little moment. The symptoms which our author describes will, in these cases, present themselves, and we shall have as constantly a deposit of oxalate of lime in the urine. Since, therefore, we comprehend distinctly what is to be included under the term oxaluria, and since, when one physician speaks to another of oxaluria he is not likely to be misunderstood, we can have no great objections to the term. It certainly hints at the presence of oxalic acid in the body, and this we consider as the real central evil in most of the cases described as oxaluria with dyspepsia.

After tracing the history of the subject, Dr. Bird declares, as regards the importance of deposits of oxalate of lime, that they are of more frequent occurrence than those of earthy phosphates—a statement which is certainly true in this city to a remarkable extent.

The next section treats of the microscopic and chemical examination of the oxalate of lime, and does justice to the careful essay of Dr. Bacon, of Boston. As regards the size of the crystals of oxalate of lime, we

* Ure, Professor Booth, etc.

would refer our readers to a short paper in the *Medical Examiner*, by Dr. Francis Lewis, who is of opinion that this deposit is very common in the urine of the black race, and that in them the crystals are of unusual magnitude, as his measurements show. No one whom Dr. Bird quotes has been able to obtain large and perfect crystals of the oxalate similar to the octohedrals found in the urine, nor does he mention the only ready mode of forming them. Two German observers have succeeded in producing very interesting results with other substances than the oxalate, by causing them to unite very slowly and gradually. We have imitated their processes in part, and have succeeded in obtaining good octohedra of oxalate of lime by placing a solution of nitrate of lime or of chloride of calcium in one vessel, and a weak solution of oxalic acid in another, a bent tube of a V shape, inverted, being filled with water and inserted in both solutions. The two fluids gradually permeated the water, and after a few weeks, fine crystals of an octohedral form were frequently obtained. Just before this, Dr. Jones, of Georgia, also procured very fine crystalline varieties of this salt, by allowing oxalic acid and soluble lime salts to mingle slowly, by permeation, through the tissues of living animals. We have found that similar and very beautiful results may be arrived at by placing the two substances on different sides of a dead membrane and allowing endosmotic currents to occur. By this means many substances which usually appear as mere granular precipitates may be obtained in well-formed crystals.

The origin of oxalate of lime in the urine is discussed by our author with remarkable acuteness. It has been doubted that oxalic acid could find its way from the stomach or from the tissues, when generated there, through a fluid like the blood containing lime salts. This doubt is due to the fact that oxalic acid, outside of the body, always seizes upon any lime which may be present, and falls as an insoluble oxalate of that base. This doubt was aided by the experiments of Scherer and others, who have discovered that oxalate of lime, *i.e.*, oxalic acid, is sometimes generated in urine by a process of fermentation. It is uncertain, however, what element of the urine undergoes destruction to produce the acid in question. Against all this, it may be urged that, when oxalic acid is taken in food, as in tomatoes, etc., it does reach the urine. The proof is absolute; and, if it can pass from the stomach to the urine, it can also pass from the tissues to that excretion. In the next place, Dr. Garrod has found the oxalate in blood. Now, whether it be due to a metamorphosis of tissue, or of the excess of nutritive matter in the blood, or to a decomposition of uric acid, we still have this unsolved difficulty as to the passage through the system of an acid which has so obstinate a tendency to form an insoluble salt of lime.

The assumption of Dr. Schmidt, of Dorpat, that the oxalate exists in the blood in the form of a soluble triple compound with albumen, has no kind of foundation. Dr. Bird, indeed, states that such a compound exists in yeast-cells, and that their decomposition sets it free. More probably the oxalic acid thus set at large is a result of the putrefactive changes in the yeast, and we are, therefore, still in the dark as regards this matter. So far as we can decide, our author regards all persistent deposits of oxalate of lime as evidence of a departure from health. Considerable experience has taught us how nearly correct are these views as stated in the following paragraph:—

"It is impossible to connect any definite set of symptoms with *all* cases in which the oxalate of lime appears in the urine; indeed, persons will often go about their ordinary duties, in apparently fair health, for a long time, and yet be constantly excreting the oxalate of lime. In consequence of this, some persons have actually affirmed that oxalate of lime has no relation with any pathological state of system, and its appearance in the urine is of no consequence. This opinion can result from very limited experience alone; indeed, I am not sure that Lehman, valuable as is his opinion as a chemist, has any claim on our confidence as a practical physician, and he is chiefly referred to as advocating the erroneous view in question."

Dr. Bird divides his cases into *a.* oxaluria with excess of urea and extractive matter in the urine; and *b.* oxaluria unattended with such excess.

We might include under this latter head all cases where the presence of oxalate is due to a diet containing the acid in question. The first class of cases *(a.)* has, according to Lehman, no existence. Our author describes them as exhibiting a set of symptoms, among which irritative dyspepsia, hypochondriasis, etc., are the most marked. We also are equally sure that such instances of general derangement of health, with such symptoms as Bird describes, do often occur, and that the presence of oxalate in the urine is, in a certain class of them, a notable symptom, both as pointing to a disease of assimilation, and to the cause of the local urinary annoyances, which so often co-exist. We are as sure that the presence of urea in excess is in them no constant symptom.

In considering, then, class *b.*, we are called upon to say whether or not oxalate of lime can be considered as a normal urinary constituent. Even admitting, with Scherer, that it may be due to a peculiar fermentation of the urine, we are loathe to believe that a salt which is present only in an insoluble crystalline state, can be regarded as a normal urinary ingredient. It is in vain to add that it is found so often in the secretion of persons in full health, since its constant presence in any urine may give rise to a calculus. Indeed, this most insidious class of cases has been quite overlooked by our author. We have again and again diagnosed the presence of this substance as a renal calculous formation, where no sign of disease existed, except the pain in the back, and the slight urinary deposit. Such cases are very common, for some reason which we do not comprehend, in the rotten lime-stone districts of this country. It is absurd, therefore, to neglect the fact of the existence of oxalate of lime in the urine, upon the presumption of its frequency entitling it to be termed otherwise than abnormal.

As regards the treatment, we feel sure that no one will consider Dr. Bird's cases as satisfactory in the way of absolute cures. All the forms of oxaluria are obstinate, when once established. It is not enough to state that now the urine was natural, or that the oxalate had disappeared, or that such a one was convalescing, in order to prove the permanent value of the means or remedies employed. In fact, the final result of our author's cases may well be a matter of doubt; and, even upon his own showing, the most of them were merely aided, and not fully relieved. For some time back, our own experience has dissatisfied us with the special treatment by acids, as recommended by Prout, upon what we incline to consider rather more than doubtful grounds; since, however, we shall shortly treat this subject in another shape, we shall here urge it no further.

The pathology of the earthy salts, is the title of the next chapter. Very little has been done for this matter since our author's last edition; and, in fact, without some new and striking discoveries, it would be difficult to improve upon Dr. Bird's account of the earthy salts.

The description next given, of pigmentary deposits in urine, is most curious and interesting. Dr. Bird distinguishes as abnormal products, the following pigments:—

Cyanourine,
Uroglaucine,
Indigo,
Prussian Blue,
Melanourine and Melanic Acid, with a few others, which are gray, or black, and, as yet, unnamed. The first and third of these pigments are in suspension in the urine, the second is in minute granules, and the third is held dissolved. As to the origin of these strange compounds, we may state that the first two are conjectured to be due to some metamorphic change in albumen. Indigo passes into the urine when given internally, but Hassall describes a blue pigment, in Bright's disease, which he thinks is indigo formed within the body. He is merely describing the uro-glaucin of Heller. The sesqui-ferro-cyanide of iron—Prussian blue—occurred in a boy who had swallowed ink. It is said that this deposit may be produced by giving to a patient, who has been taking iron, a few doses of ferro-cyanide of potassium. This appears to us quite possible, since Bernard has shown that iron may be so combined with the albumen of the blood as to be unaffected by the presence of the cyanide of potassium, until both appear in an acid fluid, as urine, or gastric juice; when at once the characteristic reaction takes place and the blue precipitate falls.

Perhaps the most important chapter in the book is that upon non-crystalline organic deposits, p. 338. In it the student is taught the diagnostic value and mode of detecting blood, pus, mucus, epithelia, spermatozoids, torula, kiesteine, albumen, sugar, etc.

A portion of the chapter describes the appearance in the urine of the various elements of the blood. Here we are struck with the difficulty of annotating the text, and yet leaving it substantially unaltered. Even the plates are useless here, because none but the most admirable delineations on steel, or copper, could convey to the eye correct representations of the peculiarities which enable us to tell one kind of tube cast from another. We miss, also, a distinct statement of *all* the circumstances which cause albumen to appear in urine. Some of these, indeed, are alluded to, but we have no discussion as to the absolute diagnostic value of the fibrin casts, under this, or that accompanying group of symptoms. This, the most important part of the volume, remains the worst. Under the headings pus and mucus, we have a few paragraphs devoted to the consideration of treatment. Here, where we should have had a clear and full appreciation of remedies, or else a reference of the whole matter to the monographs which treat of it, we get only a meagre criticism of some of the means employed, with over-praise, as usual, of pareira, and no mention at all of counter-irritants. As regards the local treatment of cystorrhœa, Dr. Bird advocates injections of warm water, and elsewhere, of dilute nitric acid, speaking of no other fluids as available for injecting the bladder. Uva ursi, chimaphila, buchu, etc., we are inclined to believe, are best given in the common form of infusions, hot or cold; and we think that the novel forms of fluid extracts, etc., are not so usefully active.

We are astonished, again, at the meagre details in regard to the various forms of epithelia, which should have been delineated distinctly, as they appear, in different parts of the urinary tracks. It is hardly right to refer the student to Dr. Beale's work on the microscope for what ought to be here.

The section on sugar urine, etc., is better done. Besides the old copper test, (Traumer's,) and the potash test, (Moore's,) we are told of bichloride of tin, (Mauméne,) of chromate of potash, (Harsley,) etc., as new tests. That of Mauméne we have found unsatisfactory. A mixture of sulphuric acid and bichromate of potassa, is said by Luton, to be the best test. The fermentation test is, beyond doubt, the least liable to suspicion, and is only inconvenient when a rapid result is required.

The "physiological and pathological origin of sugar" we should like to copy entire, did space allow of it. It is true that, a l'Anglaise, we hear so much of Dr. Pavy, who has really done something, and so little of Bernard, the parent of the whole subject as it now exists, that we fancy Dr. Pavy must shrink from such undue prominence. As a result, however, of his experiments, and those of others, it is stated that the liver forms a peculiar sugar, which disappears, partly in the lungs, and more completely in the tissues; never being entirely absent, except in the blood of the capillaries of the chylopoietic viscera. Now, in order that the sugar shall undergo destruction in the blood, it is stated that there must be, 1. Presence of molecular change; 2. Exposure to atmospheric air, or oxygen; 3. Alkaline condition of blood. The first essential is but lamely made out in the editor's statement, and does not do justice to the original observer. The remaining essentials are better stated. Starting with these, however, our editor—for we presume this section to be his work—proceeds to examine the therapeutical indications and the pathological sources of sugar. Possible it is that we are nearer the light on this matter than we were, but we must confess that, in despite of what we have learned, we do yet but grope with most uncertain steps. We have knowledge; we wait for wisdom.

We are pleased to see that our author adhered to his opinions as to the value of kiesteine as a *corroborative* test of the existence of the pregnant state. We are surprised that it is so rarely looked for as a sign of this condition, and believe that, like the larger part of the study of urine in diagnosis, it is neglected by the mass of the profession. Since the publication of Dr. Bird's first edition, the value of this test has been made more conspicuous, in despite of several papers, chiefly by Continental observers, who deny to it any value. We find no reference, by author or editor, to Dr. Kane's most admirable paper, or to the essays of Drs. McPheeters, Perry, and Elliott—all of them American writers.

Dr. Bird's book winds up with his well known original observations upon the therapeutics of the kidney, and upon the use and value of depurative and alterative agents. The very nature of this portion of the volume renders it difficult to do more than merely refer to it, as being in every way worthy of its author. We would especially call the attention of our confrères in malarious districts to the author's remarks upon renal depuration in the class of diseases which fall most under their care.

Notwithstanding the defects of the volume before us, we cannot close this review without characterizing this book as the best of its class.

It has, indeed, been feared that the increase in the number of books devoted to special subjects, or parts of practical medicine, would have a tendency to produce mere specialists, and that we are in some danger of too great a subdivision of medical labor. We are not aware, however, that this has been the case in the medical profession here, and, in fact, it is hardly likely to be so, since our cities are not large enough to support regular practitioners who attempt to confine themselves to particular kinds of business. Meanwhile, it is certainly useful to find the information upon any one subject condensed into a convenient volume, where knowledge upon a given point may easily be obtained. We have no doubt that any physician becomes a better general practitioner by making himself minutely acquainted with some particular branch of medical or surgical practice. Such a physician, in truth, was Golding Bird, and we are confident that his would be a safe example to set before those who are starting in their professional careers.

